# CIITA-Driven MHC Class II Expressing Tumor Cells as Antigen Presenting Cell Performers: Toward the Construction of an Optimal Anti-tumor Vaccine

**DOI:** 10.3389/fimmu.2019.01806

**Published:** 2019-07-30

**Authors:** Roberto S. Accolla, Elise Ramia, Alessandra Tedeschi, Greta Forlani

**Affiliations:** Laboratories of General Pathology and Immunology “Giovanna Tosi”, Department of Medicine and Surgery, School of Medicine, University of Insubria, Varese, Italy

**Keywords:** MHC-II, CIITA, CD4+ TH cells, APC, tumor vaccines

## Abstract

Construction of an optimal vaccine against tumors relies on the availability of appropriate tumor-specific antigens capable to stimulate CD4+ T helper cells (TH) and CD8+ cytolytic T cells (CTL). CTL are considered the major effectors of the anti-tumor adaptive immune response as they recognize antigens presented on MHC class I (MHC-I) molecules usually expressed in all cells and thus also in tumors. However, attempts to translate in clinics vaccination protocols based only on tumor-specific MHC-I-bound peptides have resulted in very limited, if any, success. We believe failure was mostly due to inadequate triggering of the TH arm of adaptive immunity, as TH cells are necessary to trigger and maintain the proliferation of all the immune effector cells required to eliminate tumor cells. In this review, we focus on a novel strategy of anti-tumor vaccination established in our laboratory and based on the persistent expression of MHC class II (MHC-II) molecules in tumor cells. MHC-II are the restricting elements of TH recognition. They are usually not expressed in solid tumors. By genetically modifying tumor cells of distinct histological origin with the MHC-II transactivator CIITA, the physiological controller of MHC-II gene expression discovered in our laboratory, stable expression of all MHC class II genes was obtained. This resulted in tumor rejection or strong retardation of tumor growth *in vivo* in mice, mediated primarily by tumor-specific TH cells as assessed by both depletion and adoptive cell transfer experiments. Importantly these findings led us to apply this methodology to human settings for the purification of MHC-II-bound tumor specific peptides directly from tumor cells, specifically from hepatocarcinomas, and the construction of a multi-peptide (MHC-II and MHC-I specific) immunotherapeutic vaccine. Additionally, our approach unveiled a noticeable exception to the dogma that dendritic cells are the sole professional antigen presenting cells (APC) capable to prime naïve TH cells, because CIITA-dependent MHC-II expressing tumor cells could also perform this function. Thus, our approach has served not only to select the most appropriate tumor specific peptides to activate the key lymphocytes triggering the anti-tumor effector functions but also to increase our knowledge of intimate mechanisms governing basic immunological processes.

## Introduction

In recent years, tumor immunology has witnessed a dramatic development mostly due to the possibility of applying the acquired knowledge in the field to the development of concrete and realistic approaches to fight cancer. The interest of many investigators has been concentrated mainly on ways to activate and maintain those effector cells of adaptive immunity that are believed be the major actors in eliminating the tumor cells, the CD8+ cytolytic T cells (CTL). This was justified by the fact that CTL recognize directly the tumor cells *via* their specific receptors (TcR) directed against “tumor antigens” [here defined as peptides derived from both overexpressed or mutated (neoantigens) proteins in the tumor], presented by MHC class I (MHC-I) molecules of tumor cells (1). At the variance with MHC class II (MHC-II) molecules that are constitutively expressed only in few cell types (2), MHC-I molecules are expressed, with few exceptions, in all cell types including tumor cells (3). Moreover, the intracellular pathway through which MHC-I molecules are loaded with peptides favors the binding of peptides from endogenously synthesized proteins (4, 5), as potential tumor antigens are. Unfortunately, CTL suffers of important extrinsic and intrinsic limitations in the fight against tumors. Often the tumor cells down-regulate their MHC-I expression to elude recognition by the CTL (3, 6–8); moreover tumor cells secrete in the tumor microenvironment suppressive mediators that limit the functional activity of CTL (9). Finally, and importantly, maturation, proliferation and functional activity of CTL require the continuous support of CD4+ T cells (T helper cells or TH) and this makes TH cells the master officers and the regulators of all adaptive immune responses (10, 11). Thus, the efficacy of the adaptive immune response against the tumor is strongly conditioned by the initial priming and activation of TH cells. To become fully active, TH cells must recognize antigens, including tumor antigens, via their TcR that interact with the antigen only if it is presented within the context of MHC-II molecules expressed on the surface of professional antigen presenting cells (APC), mainly dendritic cells (DC) and macrophages. At variance with MHC-I, loading of peptides on MHC-II molecules preferentially takes place in endosomal compartments (4), rich of degraded products from endocytosed external materials. Hence, it is believed that MHC-II molecules cannot present peptides derived from the processing of endogenously synthesized molecules. As mentioned above, due to their relatively restricted tissue distribution MHC-II molecules are not expressed on the majority of tumor cell types. For all these reasons, tumor cells would be prevented to stimulate TH cells and consequently to initiate the cascade of event leading to anti-tumor effector functions. The inability of tumor cells to trigger TH cells has contributed to substantiate the immunological dogma, verified for a wide variety of antigens, including pathogens, that tumor antigens could trigger the response of TH only if endocytosed, processed and presented by professional APC (12, 13). However, while for pathogens the mechanism of phago-endocytosis, digestion, processing, and presentation on the MHC-II molecules by professional APC is part of the normal physiology to eliminate the non-self external aggressors, the same is not true for tumor cells as in general these cells are not phagocytosed and degraded by APC. Thus, processing and presentation of putative immunogenic tumor antigens is strongly limited to tumor cell debris and possibly secreted tumor cell products that APC can capture in the tumor microenvironment. It is clear that in this condition the potential repertoire of tumor antigens that professional APC can process and expose via their MHC class II molecules is relatively limited both in quality and in quantity.

## Re-orienting the Focus

On the basis of the above considerations, it was not so surprising that attempts to translate to clinics vaccination protocols based only on tumor-specific MHC-I-bound peptides resulted in very limited, if any, success (7). In our opinion the failure of this vaccination attempts was mostly due to inadequate triggering of the TH arm of adaptive immunity. In this review, we focus on a novel strategy of anti-tumor vaccination established in our laboratory and based on the persistent expression of MHC-II molecules in tumor cells. Our approach started by asking a relatively naïve question: *should tumor cells have the possibility to express in a “physiological way” MHC-II molecules, would they be capable to process and present putative tumor antigens, and would they even have the capacity to trigger naïve CD4*+ *TH cells specific for tumor?*

## Canonical MHC Class II Expression in Tumor Cells Can Result in Triggering of Protective Anti-Tumor Immune Response *in vivo*

Although, MHC-II molecules can present preferentially peptides originated from protein processing in endosomal compartments and thus derived from exogenously endocytosed material, endogenous proteins could also access the MHC-II pathway of antigen presentation, as demonstrated by previous important studies (14–16) and peptides of these proteins could be recognized and serve as immunogens for TH cell triggering (17, 18). On this ground, we hypothesized that tumor cells, modified to express MHC-II molecules in an appropriate way, could present their own tumor antigens in a MHC-II-restricted fashion to tumor-specific TH cells.

As mentioned above, normally, tumor cells do not express MHC-II genes constitutively because this expression is developmentally regulated and restricted to few cell types. Nevertheless, a vast array of cell types can transiently express MHC class II genes after induction with immune cytokines, particularly IFNγ (19). Both constitutive and inducible MHC class II gene expression are under the control of the MHC class II transcriptional activator encoded by the AIR-1 locus discovered in our laboratory (20–23) and also designated CIITA (24). CIITA regulates also the expression of other fundamental genes necessary for MHC-II transport to endosomal compartments and loading of peptides, including the invariant chain (In chain) and DM (25–28). When experiments were performed to stably express CIITA in both human and mouse tumor cells, we could demonstrate the constitutive expression of MHC-II genes and corresponding molecules and, importantly, the acquisition of antigen processing and presentation to primed TH cells (29). These findings were the ground to verify *in vivo* the hypothesis that MHC-II positive tumor cells could be specifically recognized by the host immune system and establish a protective immune response. Indeed, we could demonstrate that CIITA-transfected tumor cells of distinct histological origin can be efficiently rejected or strongly retarded in their growth when injected into immunocompetent syngeneic mice (30, 31). Importantly, capacity to reject the tumors and/or strongly retard their growth was directly related to the amount of CIITA-driven MHC class II molecules expressed on the cancer cell surface (30–32). Furthermore, it was shown that CIITA-tumor vaccinated mice develop an anamnestic response not only against the CIITA-transfected tumor but, most importantly, against the parental tumor leading to a very efficient rejection of the parental tumor as well. The expression of MHC class II molecules driven by CIITA was an obligatory requirement to induce the anamnestic protective response against the parental tumor, and this received confirmation also by experiments using as a vaccine non-replicating CIITA-transfected tumor cells (33).

Careful analysis of the mechanisms of protection highlighted several crucial aspects. First, enduring immunity was generated in CIITA-tumor vaccinated as shown by the fact that these mice remained immune from further challenge with parental tumor cells for many months. Moreover, anti-tumor effector mechanisms were specifically mediated by CD4+ TH cells and CTL, since elimination of these cell subpopulation *in vivo* by injecting anti-CD4 or anti-CD8 specific antibodies, abrogated the capacity of the animals to generate protective immunity after administration of CIITA-tumor cells. On the other hand, elimination of B cells or NK cells did not affect the capacity of the animals to reject CIITA-tumor cells. Finally, the crucial importance of CD4+ TH cells as key players in the generation of protective anti-tumor immunity was substantiated by adoptive cell transfer experiments of CD4+ cells from vaccinated mice into naïve recipients and consequent acquisition of protection from tumor growth when challenged with parental tumor cells.

Cumulatively, these findings demonstrated the that the expression of MHC class II molecules driven by CIITA in tumor cells was key in triggering an adaptive and protective immunity.

These results were at variance with respect to those obtained by the group of Ostrand-Rosenberg and colleagues, who studied the function of MHC class II expression in tumors by focusing however mostly on a single tumor model, the H-2^K^ SaI sarcoma, and on MHC class II alpha-beta transfected genes, in absence of invariant chain, reaching the conclusion that class II-transfected cells could be better rejected as compared to CIITA-transfected cells (34, 35). We have extensively discussed in a previous publication (36) the immunological constraints and limitations of this approach and the consequent biological conclusions, due mostly to the fact that MHC class II molecules are highly unstable in absence of invariant chain and therefore they can hardly go to the cell surface end present antigenic peptides for appropriate recognition by CD4+ T cells (6, 37).

## The Tumor Microenvironment Switch in CIITA-Tumor Vaccinated Mice

The comparative study of the tumor microenvironment and tumor draining lymph nodes of animals injected with parental tumor or CIITA-tumor cells gave additional and crucial hints for understanding the mechanism through which CIITA-tumor cells triggered a protective immune response (32). Little infiltration composed mostly by macrophages and neutrophils, and virtually no CD4+ T cells, CD8+ T cells, and DC was observed in tumors derived from parental cells. In contrast a rapid infiltration of CD4+ T cells, followed by DC and CD8+ T cells was observed at the tumor site when mice were injected with CIITA-tumors. Interestingly the CIITA-tumor microenvironment was characterized by extensive areas of tumor cell necrosis. Furthermore, in CIITA-tumor vaccinated mice challenged with parental tumors, the number of infiltrating lymphocytes and the extension of necrotic tissue were clearly larger than those found in naïve mice injected with CIITA-tumor cells (31).

**Figure 1:**
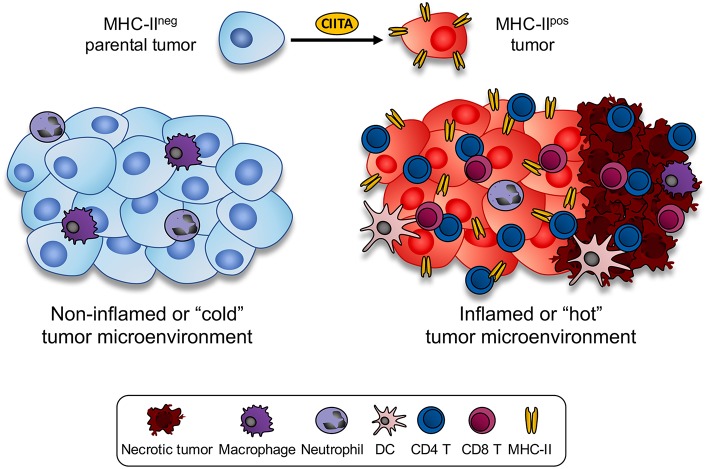
Expression of CIITA-driven MHC-II expression in tumor cells drastically modify the histology of the tumor microenvironment. MHC class II negative mouse tumors of distinct histologic origin and H-2 genotype (left side) are very little infiltrated by blood-derived cells (cold or non-inflamed tumor). In the mouse tumor models analyzed in our studies, scarce infiltration of neutrophils and monocyte-macrophages was detected *in vivo* in the microenvironment of parental tumors. Upon stable transfection with CIITA and consequent expression of MHC class II molecule, tumors became rapidly infiltrated by CD4+ T cells, followed by CD8+ T cells and only later by dendritic cells and macrophages (inflamed or hot tumor). As result of the intense lymphocyte infiltration, large areas of tumor necrosis were generated. Thus, the tumor microenvironment was drastically modified by the CIITA-driven MHC class II expression in the tumor cells.

The above histological aspect in parental tumor-injected vs. CIITA-tumor injected mice was indeed representative of what is generally described as a “non-inflamed or cold” vs. an “inflamed or hot” tumor microenvironment, respectively (38, 39). Thus, forcing the “physiological” expression of MHC-II molecules by transfecting CIITA into tumor cells resulted in a dramatic modification of the tumor microenvironment which was associated to specific tumor rejection and/or strong retardation of tumor growth (Figure 1). Within this frame it is tempting to speculate that in spontaneous tumors characterized by an inflamed microenvironment, tumor infiltrating CD4+ as well as CD8+ T cells by actively secreting IFNγ may transiently induce CIITA expression and consequently MHC class II gene expression in naïve tumor cells resulting in further recognition and killing of the tumor. Additionally, tumor-draining lymph nodes of mice vaccinated with CIITA-tumor cells showed a more polarized TH1-type phenotype with respect to a rather polarized TH2-type phenotype observed in similar lymph nodes of mice injected with parental tumor cells. It should be underlined the strong anti-tumor T cell immunity was not accompanied by manifestations of autoimmunity, suggesting tumor-specific and not self-antigens were the target of the observed anti-tumor response. The subversion of the tumor microenvironment affected also the number CD4+/CD25+ regulatory T cells (Tregs) in draining lymp nodes. It is generally accepted that Tregs play an important role in regulating the activity of CD4+ TH cells. In tumor-bearing, hosts is often observed an increase in number and corresponding function of Tregs (40). In our tumor model, we found an increase in draining lymph nodes of parental tumor-bearing mice not paralleled, however, by a functional increase in suppressive function *in vitro* and *in vivo* (41). On the other hand in CIITA-tumor vaccinated mice, the number of Tregs was clearly reduced and comparable to the number of naïve animals (33). This led us to conclude that vaccination with CIITA-tumor cells affected also a crucial component of the regulatory circuit, the Tregs, by preventing their increase in number in the tumor microenvironment and in so doing facilitating the triggering and persistence of anti-tumor CD4+ TH cells (36).

## CIITA-Driven MHC-II Expressing Tumor Cells are the Major APC *in vivo*

Cumulatively, the above described studies clearly demonstrated that CIITA-driven MHC-II expressing tumor cells are strongly recognized *in vivo* and trigger tumor specific CD4+ TH cell responses that are protective against subsequent rechallenge with parental tumors. Nevertheless, they did not formally prove that CIITA-tumor cell could function as classical APC in triggering the priming of naïve tumor antigen-specific TH cells. The possibility remained that priming of naïve TH cells could be still mediated by professional APC capturing of MHC-II-peptide complexes derived from dying CIITA-tumor cells or from cellular debris.

The final demonstration that CIITA-mediated MHC-II expressing tumor cells could indeed function as classical APC came recently by using a transgenic mouse model, in which professional APC can be transiently deleted. These transgenic C57BL/6 H-2^b^ mice, designated CD11c.DTR, carry the diphteria toxin receptor under the control of the CD11c promoter, which is strongly expressed in DC. Thus, in these animals dendritic cells can be conditionally deleted by administration of diphteria toxin (42). Two highly tumorigenic MHC-II-negative C57BL/6 H-2^b^ tumor cell lines, MC38 colon carcinoma and LLC Lewis lung carcinoma, were stably transfected with CIITA and selected for expression of MHC class II molecules. When injected *in vivo* in CD11c.DTR mice both these CIITA-tumors were rejected or strongly retarded in their growth. Importantly the same behavior was observed after treatment with diphtheria toxin to eliminate DC (43).

The mice rejecting the tumor were immune to MHC-II-negative parental tumors and their CD4+ TH cells protected naïve H-2^b^ C57Bl/6 mice in adoptive cell transfer experiments. To exclude that additional professional APC like macrophages, in absence of DC could serve as main subpopulation to prime tumor-specific naïve CD4 T cells, CD11c.DTR transgenic mice were treated with liposomal Clodronate, a compound that is selectively engulphed by macrophages. Upon phagocytosis, liposomal Clodronate kills the cells by apoptosis (44). Interestingly, in the spleen liposomal Clodronate is engulphed by and kill quite selectively the marginal zone and the metallophilic macrophages considered the predominant APCs (45). Even after treatment with liposomal Clodronate, mice injected with CIITA-tumor cells could reject or strongly retard tumor growth with a behavior very similar to the one observed in liposomal Clodronate-untreated mice (43). Thus, CIITA-driven MHC-II positive tumor cells can perform not only antigen processing and presenting function *in vitro* at least for primed T cells of either human (29) or mouse (32) but, more importantly, they can prime *in vivo* naïve CD4+ TH cells and thus serve as *bona fide* APC to generate a strong adaptive immune response capable to protect against the tumor (43, 46).

Of relevance, recent work indicated that the MHC class II-positive H-2^d^ A20 B cell lymphoma cells expressing GFP (A20-GFP), but not the MHC class II-negative H-2^d^ 4T1-GFP mammary carcinoma cells, can indeed prime directly and be killed *in vitro* by syngeneic CD8 T cells specific for GFP, although in this particular system *in vivo* cross-priming by dendritic cells may also be required (47). These experiments underline the importance of MHC class II expression on tumors to elicit optimal antigen priming, although *in vivo* they may not apply to all tumor histotypes.

Collectively, our findings have not only practical but also conceptual consequences because they challenge the widely accepted view of the exquisite supremacy of DC and, to lesser extent, macrophages to serve as sole APC for priming antigen-specific naïve CD4+ TH cells (9). Whether CIITA-driven MHC class II expressing tumor cells may also spontaneously acquire or be endowed in part with phagocytotic function and thus eat the other dead tumor cells and cross-present their tumor antigen to the naïve lymphocytes just like human immature DCs, remains to be investigated.

Another interesting consideration derived from the above results relates to the genetic characteristics of C57BL/6 H-2^b^ and their transgenic derivative CD11c.DTR mice. These mice express only one subclass of MHC class II molecule, the I-A molecules because of a defect of the Eα gene (48). Thus, not only tumor cells of distinct genetic background and distinct histotype origin can become immunogenic when expressing CIITA-driven MHC class II molecules (31, 43) but they can also do so by presenting relevant and sufficient tumor derived-peptides within a single MHC-II restricting element, the IA molecule. Very similar results were obtained by other investigators in a pancreatic ductal adenocarcinoma model of C57BL/6 H-2^b^ (49).

The capacity of CIITA-dependent MHC class II expressing tumor cells to serve as APC *in vivo* raises the question of whether these cells possess or acquire the expression of co-stimulatory molecules, such as B7.1 (CD80) and B7.2 (CD86) that may serve as “signal 2” in triggering antigen-specific naïve TH cells upon interaction with CD28 (50), as previous studies of another group has shown that prevention of tumor growth *in vivo* of CIITA-modified tumor cells in a distinct model of mammary carcinoma in H-2^q^ model required also expression of CD80 (51). We found that MC38 and LLC tumor cells do not express CD80 and CD86 costimulatory molecules and this phenotype is not modified by CIITA expression. Thus, either CIITA-tumors do not need necessarily accessory molecules to perform their APC function *in vivo*, or other accessory molecules are involved to provide the second signal, or tumor-specific, and possibly organ-specific constraints limit the immune stimulating function of CIITA-driven MHC class II expressing tumor cells.

This important issue should certainly deserve detailed investigation in the future.

As outlined earlier and in relation to the peculiar modification of the tumor microenvironment generated by CIITA-driven MHC class II positive tumor cells, our studies raise another relevant question related to the anatomical location in which the anti-tumor immune response against CIITA-modified cancer cells takes place. It is generally assumed that TH cell priming mediated by professional APC, namely DC, takes place in the lymph nodes, where DC that have captured and processed the antigens in the periphery migrate and present antigenic peptides within the context of MHC-II molecules (12). As the tumor microenvironment was drastically modified in presence of CIITA-tumor cells, with a profound change both in number and compartmentalization of the leukocyte infiltration (32) we may speculated that it could be the ideal site for the formation of ectopic lymphoid-like structures or tertiary lymphoid organs (TLO), neoformations that are often detected in chronic inflamed tissues and in tumor tissues (36, 52). TLO share many characteristics with lymph nodes associated with the generation of an adaptive immune response (53). If this will be confirmed in future studies, tumors cells expressing MHC class II molecules not only act as APC for priming naïve tumor-specific CD4+ T cells but also perform APC activity ectopically with respect to the canonical site represented by the lymph nodes.

## From the Bench to the Bedside: the Construction of an Optimal Anti-Tumor Therapeutic Vaccine… and Beyond

**Figure 2:**
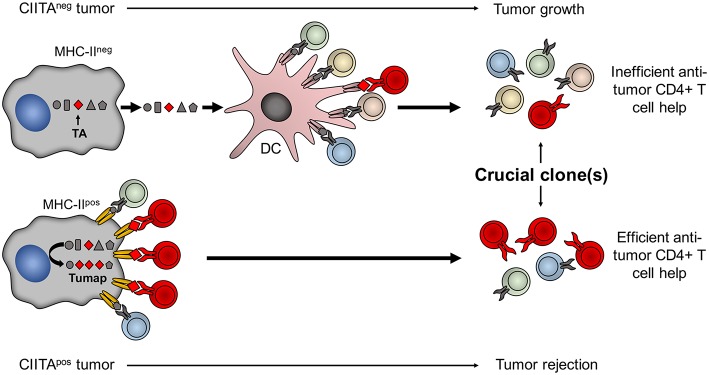
Tumor cells expressing CIITA-driven MHC-II molecules are potent surrogate APC to prime relevant tumor-specific TH cells. The MHC class II-bound tumor peptidome (Tumap, red symbols) derived from CIITA-driven MHC-II expressing cells is highly enriched of tumor-specific epitopes (Lower part) as compared to the one of classical APC, such as dendritic cells (DC), that may capture tumor antigens (TA) only after phagocytosis of dying MHC-II-negative tumor cell debris (Upper part). As a result, in CIITA-tumors, MHC-II-tumor peptide complexes efficiently stimulate and amplify higher number of tumor-specific TH cell clones (in red) to generate a strong immune response capable to reject the tumor (Lower part). On the contrary, classical APC do not efficiently select sufficient tumor-specific peptides from MHC-II-negative tumor cells to be presented within the context of their MHC-II. As a consequence DC cannot efficiently prime tumor-specific TH cell clones and tumor takes off and grows (Upper part).

A major corollary of the studies related to the high *in vivo* immunogenicity of CIITA-driven MHC-II expressing tumors is that MHC class II molecules should be loaded with sufficient quantity of tumor specific peptides, derived from either overexpressed or mutated genes, to generate an efficient functional triggering of tumor-specific CD4+ T cells, an event that we have defined as Adequate Antigen Availability (AAA) (54). Thus, these cells can be instrumental to identify the key tumor antigens which may serve to develop new generation anti-tumor vaccines (Figure 2).

This strategy has indeed been applied recently by a European Consortium of nine institutions, including our laboratory (the Hepavac Consortium, www.hepavac.eu), as part of the construction of an innovative vaccine against human hepatocarcinomas (HCC). HCC was selected because ranks sixth in terms of incidence but fourth in term of deaths/year worldwide (GLOBOCAN 2018, http://gco.iarc.fr/). Given the current lack of available effective treatments, the overall prognosis for patients with HCC is poor with a dismail 5-year survival of <25%, making the disease a highly important and relevant target for the development of innovative therapies (55).

By using a well-established experimental protocol and purification platform (56), the relevant MHC II-bound tumor specific peptides were selected from CIITA-driven MHC-II expressing human HCC cells. These peptides, along with a number of highly specific HCC MHC-I-bound tumor peptides, contributed to the formation of a peptide cocktail to be used as the first multi-epitope, multi-target, and multi-allele cancer vaccine against HCC aimed at stimulating both CD4+ and CD8+ T cells. This vaccine is, at present, in a phase I/II clinical trial whose results on safety, tolerability, and immunogenicity (primary endpoints) and possibly overall survival (secondary endpoint) are expected by the end of 2019.

In studying patient's HCC tumor tissues as well as normal liver tissues, we observed two important features that bear relevance not only for applying profitable vaccination approaches as the one described here but also to better understand old and recent observations on the immunologically tolerant environment of the liver (57, 58). The first important observation was related to the expression of MHC-I and MHC-II in liver cells. While both these molecules were virtually absent in normal liver cells, MHC-I cell surface molecules were expressed at very high level in HCC cells (59). This of course was relevant to purify the MHC-I tumor peptidome and select the appropriate peptides for the vaccine compositions. The second important observation was that MHC-II expression, instead, remained silent in HCC cells both *in vivo* and in patients' derived tumor cell lines. Importantly MHC-II expression could not be rescued even by treatment with IFNγ, the most potent inflammatory cytokine that induces MHC-II expression indirectly via the primary transcriptional activation of CIITA (19). In depth analysis of the molecular mechanism responsible of this finding demonstrated that the CIITA promoter IV, the specific promoter activated by the IFNγ (60), was silenced by hypermethylation of its sequence and thus rendered developmentally unresponsive in liver cells (59). This finding may have important effects on the interpretation of the tolerogenic environment of the liver, because the impossibility to express MHC-II molecules by liver cells, continuously in contact with massive concentrations of antigenic materials derived from the digestive tract, would prevent accidental co-participation of these cells to APC function and activation of immune system against potential food antigens as well as other antigens including self antigens.

Thus, as it was the case for the discovery of the surrogate APC function of CIITA-driven MHC-II expressing tumor cells, we also believe that unveiling the tissue constraints at the basis of CIITA-driven MHC class II expression or non-expression may serve to better understand important aspects of basic immunology.

## Author Contributions

GF and RA conceived and wrote the paper. All authors revised and approved the final manuscript.

### Conflict of Interest Statement

The authors declare that the research was conducted in the absence of any commercial or financial relationships that could be construed as a potential conflict of interest.

## References

[1] Boon T Cerottini JC Van den Eynde B Van der Bruggen P Van Pel A Tumor antigens recognized by T lymphocytes. Annu Rev Immunol. (1994) 12:337–65. 10.1146/annurev.iy.12.040194.002005 8011285

[2] Daar AS Fuggle SV Fabre JW Ting A Morris PJ. The detailed distribution of MHC Class II antigens in normal human organs. Transplantation. (1984) 38:293–8. 10.1097/00007890-198409000-00019 6591602

[3] Garcia-Lora A Algarra I Garrido F. MHC class I antigens, immune surveillance, and tumor immune escape. J Cell Physiol. (2003) 195:346–55. 10.1002/jcp.10290 12704644

[4] Rock KL Reits E Neefjes J. Present yourself! By MHC class I and MHC class II molecules. Trends Immunol. (2016) 37:724–37. 10.1016/j.it.2016.08.010 27614798 PMC5159193

[5] Kelly A Trowsdale J. Genetics of antigen processing and presentation. Immunogenetics. (2018) 71:161–70. 10.1007/s00251-018-1082-2 30215098 PMC6394470

[6] Kageshita T Hirai S Ono T Hicklin DJ Ferrone S. Down-regulation of HLA class I antigen-processing molecules in malignant melanoma: association with disease progression. Am J Pathol. (1999) 154:745–54. 10.1016/S0002-9440(10)65321-7 10079252 PMC1866429

[7] Rosenberg SA Yang JC Restifo NP. Cancer immunotherapy: moving beyond current vaccines. Nat Med. (2004) 10:909–15. 10.1038/nm1100 15340416 PMC1435696

[8] Garrido F Algarra I García-Lora AM. The escape of cancer from T lymphocytes: immunoselection of MHC class I loss variants harboring structural-irreversible “hard” lesions. Cancer Immunol Immunother. (2010) 59:1601–6. 10.1007/s00262-010-0893-2 20625726 PMC11029827

[9] Chen DS Mellman I. Oncology meets immunology: the cancer-immunity cycle. Immunity. (2013) 39:1–10. 10.1016/j.immuni.2013.07.012 23890059

[10] Germain RN Margulies DH The biochemistry and cellular biology of antigen processing and presentation. Annu Rev Immunol. (1993) 11:403–50. 10.1146/annurev.iy.11.040193.002155 8476568

[11] Accolla RS Tosi G. Optimal MHC-II-restricted tumor antigen presentation to CD4+ T helper cells: the key issue for development of anti-tumor vaccines. J Transl Med. (2012) 10:154. 10.1186/1479-5876-10-154 22849661 PMC3478985

[12] Steinman RM. Dendritic cells: versatile controllers of the immune system. Nat Med. (2007) 13:1155–9. 10.1038/nm1643 17917664

[13] Melief CJM van Hall T Arens R Ossendorp F van der Burg SH. Therapeutic cancer vaccines. J Clin Invest. (2015) 125:3401–12. 10.1172/JCI80009 26214521 PMC4588240

[14] Rudensky A Preston-Hurlburt P Hong SC Barlow A Janeway CAJ. Sequence analysis of peptides bound to MHC class II molecules. Nature. (1991) 353:622–7. 10.1038/353622a0 1656276

[15] Schimd D Pypaert M Munz C Antigen-loading compartments for major histocompatibility class II molecules continuously receive input from autophagosomes. Immunity. (2007) 26:79–92. 10.1016/j.immuni.2006.10.018 17182262 PMC1805710

[16] Kremer AN van der Meijden ED Honders MW Goeman JJ Wiertz EJHJ Falkenburg JHF. Endogenous HLA class II epitopes that are immunogenic *in vivo* show distinct behavior toward HLA-DM and its natural inhibitor HLA-DO. Blood. (2012) 120:3246–55. 10.1182/blood-2011-12-399311 22889757

[17] Jaraquemada D Marti M Long EO An endogenous processing pathway in vaccinia virus-infected cells for presentation of cytosolic antigens to MHC class II-restricted T cells. J Exp Med. (1990) 172:947–54. 10.1084/jem.172.3.947 2388037 PMC2188531

[18] Chicz RM Urban RG Gorga JC Vignali DA Lane WS Strominger JL. Specificity and promiscuity among naturally processed peptides bound to HLA-DR alleles. J Exp Med. (1993) 178:27–47. 10.1084/jem.178.1.27 8315383 PMC2191090

[19] Accolla RS De Lerma Barbaro A Mazza S Casoli C De Maria A Tosi G. The MHC class II transactivator: prey and hunter in infectious diseases. Trends Immunol. (2001) 22:560–3. 10.1016/S1471-4906(01)02003-8 11574280

[20] Accolla RS. Human B cell variants immunoselected against a single Ia subset have lost expression of several Ia antigen subsets. J Exp Med. (1983) 157:1053–8. 10.1084/jem.157.3.1053 6403646 PMC2186959

[21] Accolla RS Scarpellino L Carra G Guardiola J. Trans-acting element(s) operating across species barriers positively regulate the expression of major histocompatibility complex class II genes. J Exp Med. (1985) 162:1117–33. 10.1084/jem.162.4.1117 3862745 PMC2187858

[22] Accolla RS Jotterand-Bellomo M Scarpellino L Maffei A Carra G Guardiola J. Air-1, a newly found locus on mouse chromosome 16 encoding a trans-acting activator factor for MHC class II gene expression. J Exp Med. (1986) 164:369–74. 10.1084/jem.164.1.369 3088202 PMC2188193

[23] Guardiola J Scarpellino L Carra G Accolla RS. Stable integration of mouse DNA into Ia-negative human B-lymphoma cells causes reexpression of the human Ia-positive phenotype. Proc Natl Acad Sci USA. (1986) 83:7415–8. 10.1073/pnas.83.19.7415 3489937 PMC386728

[24] Steimle V Otten LA Zufferey M Mach B. Complementation cloning of an MHC class II transactivator mutated in hereditary MHC class II deficiency (or bare lymphocyte syndrome). Cell. (1993) 75:135–46. 10.1016/S0092-8674(05)80090-X 8402893

[25] Accolla RS Carra G Buchegger F Carrel S Mach JP The human Ia-associated invariant chain is synthesized in Ia-negative B cell variants and is not expressed on the cell surface of both Ia-negative and Ia-positive parental cells. J Immunol. (1985) 134:3265–71. 3872331

[26] Viville S Neefjes J Lotteau V Dierich A Lemeur M Ploegh H. Mice lacking the MHC class II-associated invariant chain. Cell. (1993) 72:635–48. 10.1016/0092-8674(93)90081-Z 7679955

[27] Harton JA Ting JP. Class II transactivator: mastering the art of major histocompatibility complex expression. Mol Cell Biol. (2000) 20:6185–94. 10.1128/MCB.20.17.6185-6194.2000 10938095 PMC86093

[28] Neefjes J Jongsma MLM Paul P Bakke O. Towards a systems understanding of MHC class I and MHC class II antigen presentation. Nat Rev Immunol. (2011) 11:823–36. 10.1038/nri3084 22076556

[29] Sartoris S Valle MT Barbaro ADL Tosi G Cestari T D'Agostino A. HLA class II expression in uninducible hepatocarcinoma cells after transfection of AIR-1 gene product CIITA: acquisition of antigen processing and presentation capacity. J Immunol. (1998) 161:814–20. 9670958

[30] Meazza R Comes A Orengo AM Ferrini S Accolla RS. Tumor rejection by gene transfer of the MHC class II transactivator in murine mammary adenocarcinoma cells. Eur J Immunol. (2003) 33:1183–92. 10.1002/eji.200323712 12731043

[31] Frangione V Mortara L Castellani P De Lerma Barbaro A Accolla RS. CIITA-driven MHC-II positive tumor cells: preventive vaccines and superior generators of antitumor CD4+ T lymphocytes for immunotherapy. Int J Cancer. (2010) 127:1614–24. 10.1002/ijc.25183 20091859

[32] Mortara L Castellani P Meazza R Tosi G De Lerma Barbaro A Procopio FA. CIITA-induced MHC class II expression in mammary adenocarcinoma leads to a Th1 polarization of the tumor microenvironment, tumor rejection, and specific antitumor memory. Clin Cancer Res. (2006) 12:3435–43. 10.1158/1078-0432.CCR-06-0165 16740768

[33] Mortara L Frangione V Castellani P De Lerma Barbaro A Accolla RS. Irradiated CIITA-positive mammary adenocarcinoma cells act as a potent anti-tumor-preventive vaccine by inducing tumor-specific CD4+ T cell priming and CD8+ T cell effector functions. Int Immunol. (2009) 21:655–65. 10.1093/intimm/dxp034 19395374

[34] Armstrong TD Clements VK Martin BK Ting JP Ostrand-Rosenberg S. Major histocompatibility complex class II-transfected tumor cells present endogenous antigen and are potent inducers of tumor-specific immunity. Proc Natl Acad Sci USA. (1997) 94:6886–91. 10.1073/pnas.94.13.6886 9192661 PMC21254

[35] Dolan BP Gibbs KD Ostrand-Rosenberg S. Tumor-specific CD4+ T cells are activated by “cross-dressed” dendritic cells presenting peptide-MHC class II complexes acquired from cell-based cancer vaccines. J Immunol. (2006) 176:1447–55. 10.4049/jimmunol.176.3.1447 16424172

[36] Accolla RS Lombardo L Abdallah R Raval G Forlani G Tosi G. Boosting the MHC class II-restricted tumor antigen presentation to CD4+ T helper cells: a critical issue for triggering protective immunity and re-orienting the tumor microenvironment toward an anti-tumor state. Front Oncol. (2014) 4:32. 10.3389/fonc.2014.00032 24600588 PMC3927100

[37] Bikoff EK Huang LY Episkopou V van Meerwijk J Germain RN Robertson EJ. Defective major histocompatibility complex class II assembly, transport, peptide acquisition, and CD4+ T cell selection in mice lacking invariant chain expression. J Exp Med. (1993) 177:1699–712. 10.1084/jem.177.6.1699 8098731 PMC2191043

[38] Chen DS Mellman I. Elements of cancer immunity and the cancer–immune set point. Nature. (2017) 541:321–30. 10.1038/nature21349 28102259

[39] Gujar S Pol JG Kroemer G. Heating it up: oncolytic viruses make tumors 'hot' and suitable for checkpoint blockade immunotherapies. Oncoimmunology. (2018) 7:e1442169. 10.1080/2162402X.2018.1442169 30221036 PMC6136862

[40] Sakaguchi S Naturally arising CD4+ regulatory T cells for immunologic self-tolerance and negative control of the immune response. Annu Rev Immunol. (2004) 22:531–62. 10.1146/annurev.immunol.21.120601.141122 15032588

[41] Mortara L Balza E Sassi F Castellani P Carnemolla B De Lerma Barbaro A. Therapy-induced antitumor vaccination by targeting tumor necrosis factor-a to tumor vessels in combination with melphalan Eur J Immunol. (2007) 37:3381–92. 10.1002/eji.200737450 18022863

[42] Hochweller K Striegler J Hammerling GJ Garbi N. A novel CD11c.DTR transgenic mouse for depletion of dendritic cells reveals their requirement for homeostatic proliferation of natural killer cells. Eur J Immunol. (2008) 38:2776–83. 10.1002/eji.200838659 18825750

[43] Bou Nasser Eddine F Forlani G Lombardo L Tedeschi A Tosi G Accolla RS. CIITA-driven MHC class II expressing tumor cells can efficiently prime naive CD4(+) TH cells *in vivo* and vaccinate the host against parental MHC-II-negative tumor cells. Oncoimmunology. (2016) 6:e1261777. 10.1080/2162402X.2016.1261777 28197387 PMC5283634

[44] Van Rooijen N Sanders A. Liposome mediated depletion of macrophages: mechanism of action, preparation of liposomes and applications. J Immunol Methods. (1994) 174:83–93. 10.1016/0022-1759(94)90012-4 8083541

[45] Seiler P Aichele PB Odermatt B Hengartner H Zinkernagel RM Schwendener RA. Crucial role of marginal zone macrophages and marginal zone metallophils in the clearance of lymphocytic choriomeningitis virus infection. Eur J Immunol. (1997) 27:2626–33. 10.1002/eji.1830271023 9368619

[46] Bou Nasser Eddine F Ramia E Tosi G Forlani G Accolla RS. Tumor immunology meets…immunology: modified cancer cells as professional APC for priming naïve tumor-specific CD4+ T cells. Oncoimmunology. (2017) 6:e1356149. 10.1080/2162402X.2017.1356149 29147609 PMC5674956

[47] Hammerich L Marron TU Upadhyay R Svensson-Arvelund J Dhainaut M Hussein S. Systemic clinical tumor regressions and potentiation of PD1 blockade with *in situ* vaccination. Nat Med. (2019) 25:814–24. 10.1038/s41591-019-0410-x 30962585

[48] Mathis DJ Benoist C Williams VE II Kanter M McDevitt HO. Several mechanisms can account for defective E alpha gene expression in different mouse haplotypes. Proc Natl Acad Sci USA. (1983) 80:273–7. 10.1073/pnas.80.1.273 6296871 PMC393355

[49] Ekkirala CR Cappello P Accolla RS Giovarelli M Romero I Garrido C. Class II transactivator-induced MHC class II expression in pancreatic cancer cells leads to tumor rejection and a specific antitumor memory response. Pancreas. (2014) 43:1066–72. 10.1097/MPA.0000000000000160 24987872

[50] Greenwald RJ Freeman GJ Sharpe AH. The B7 family revisited. Annu Rev Immunol. (2005) 23:515–48. 10.1146/annurev.immunol.23.021704.115611 15771580

[51] Jabrane-Ferrat N Campbell MJ Esserman LJ Peterlin BM. Challenge with mammary tumor cells expressing MHC class II and CD80 prevents the development of spontaneously arising tumors in MMTV-neu transgenic mice. Cancer Gene Ther. (2006) 13:1002–10. 10.1038/sj.cgt.7700974 16841083

[52] Aloisi F Pujol-Borrell R. Lymphoid neogenesis in chronic inflammatory diseases. Nat Rev Immunol. (2006) 6:205–17. 10.1038/nri1786 16498451

[53] Dieu-Nosjean M-C Goc J Giraldo NA Sautès-Fridman C Fridman WH. Tertiary lymphoid structures in cancer and beyond. Trends Immunol. (2014) 35:571–80. 10.1016/j.it.2014.09.006 25443495

[54] Accolla RS Tosi G. Adequate antigen availability: a key issue for novel approaches to tumor vaccination and tumor immunotherapy. J Neuroimmune Pharmacol. (2013) 8:28–36. 10.1007/s11481-012-9423-7 23224729

[55] Ulahannan SV Duffy AG McNeel TS Kish JK Dickie LA Rahma OE. Earlier presentation and application of curative treatments in hepatocellular carcinoma. Hepatology. (2014) 60:1637–44. 10.1002/hep.27288 24996116 PMC4211986

[56] Singh-Jasuja H Emmerich N Rammensee H. The Tübingen approach: identification, selection, and validation of tumor-associated HLA peptides for cancer therapy. Cancer Immunol Immunother. (2004) 53:187–95. 10.1007/s00262-003-0480-x 14758508 PMC11032959

[57] Crispe IN. Hepatic T cells and liver tolerance. Nat Rev Immunol. (2003) 3:51–62. 10.1038/nri981 12511875

[58] Makarova-Rusher OV Medina-Echeverz J Duffy AG Greten TF. The yin and yang of evasion and immune activation in HCC. J Hepatol. (2015) 62:1420–9. 10.1016/j.jhep.2015.02.038 25733155

[59] Ramia E Chiaravalli AM Bou Nasser Eddine F Tedeschi A Sessa F Accolla RS. CIITA-related block of HLA class II expression, upregulation of HLA class I, and heterogeneous expression of immune checkpoints in hepatocarcinomas: implications for new therapeutic approaches. OncoImmunology. (2019) 8:1548243. 10.1080/2162402X.2018.1548243 30723578 PMC6350839

[60] Steimle V Siegrist C Mottet A Lisowska-Grospierre B Mach B. Regulation of MHC class II expression by interferon-gamma mediated by the transactivator gene CIITA. Science. (1994) 265:106–9. 10.1126/science.8016643 8016643

